# Secular trend and risk factors of 30-day COPD-related readmission in Beijing, China

**DOI:** 10.1038/s41598-022-20884-3

**Published:** 2022-10-05

**Authors:** Jiachen Li, Lirong Liang, Siyu Cao, Hengmo Rong, Lin Feng, Di Zhang, Shuilian Chu, Hang Jing, Zhaohui Tong

**Affiliations:** 1 Department of Clinical Epidemiology, Beijing Institute of Respiratory Medicine and Beijing Chao-Yang Hospital, Capital Medical University, No. 8 Gongren Tiyuchang Nanlu, Chaoyang District, Beijing, 100020 China; 2 Department of Respiratory and Critical Care Medicine, Beijing Institute of Respiratory Medicine and Beijing Chao-Yang Hospital, Capital Medical University, No. 8 Gongren Tiyuchang Nanlu, Chaoyang District, Beijing, 100020 China

**Keywords:** Respiratory tract diseases, Epidemiology

## Abstract

Readmission due to chronic obstructive pulmonary disease (COPD) exacerbation contributes significantly to disease burden. Trend in readmission rate among COPD patients in China is not well characterized. We described the secular trend and identify risk factors of COPD-related 30-day readmission in Beijing during 2012–2017. In this retrospective cohort study, we used data from a citywide hospital discharge database in Beijing. We included patients ≥ 40 years with a primary diagnosis of COPD from 2012 to 2017. A total of 131 591 index admissions were identified. COPD-related 30-day readmission was defined as the initial admission with a primary diagnosis of COPD that occurs within 30 days from the discharge date of an index admission. Overall and annual 30-day readmission rates were calculated in the total population and subgroups defined by patient characteristics. We used multivariable logistic models to investigate risk factors for readmission and in-hospital mortality within 30 days. The overall 30-day COPD-related readmission rate was 15.8% (n = 20 808). The readmission rate increased from 11.5% in 2012 to 17.2% in 2017, with a multivariable-adjusted OR (95% CI) for annual change to be 1.08 (1.06–1.09) (*P* trend < 0.001). The upward trend in readmission rate levelled off at about 17% since 2014. The readmission rate of men was higher and increased faster than women. Comorbid osteoporosis, coronary heart disease, congestive heart failure, and cancer were associated with an increased risk of 30-day COPD-related readmission. The 30-day COPD-related readmission rate in Beijing showed an overall increasing trend from 2012 to 2017. Future efforts should be made to further improve care quality and reduce early readmissions of COPD patients.

## Introduction

Chronic obstructive pulmonary disease (COPD) is a common respiratory disease that has become a leading cause of death and disability in the world^1^. More than 80% of COPD deaths occurred in low- and middle-income countries (LMIC)^2^. In China, the prevalence of COPD in adults aged 20 years or older is 8.6%, accounting for 99.9 million people with COPD^3^. Improving disease management is a priority for such a large number of COPD patients. An important goal for COPD management is to prevent acute exacerbations^4,5^. COPD exacerbations cause hospitalization and readmission events, contributing significantly to medical expenditures^6^.

Hospital readmission rate within 30 days after discharge is a marker of health care quality. It was estimated that approximately 20% of COPD patients were readmitted within 30 days^7^. Some countries have made efforts to reduce excess readmissions^8–10^. The Chinese government implemented policies to reduce medical costs by controlling the mean length of stay. But the quality of care may be affected, and the readmission risk may increase. Consequently, the total expenditures per capita would not decline^11^. Recent data indicated that the increase in expenditures for COPD exacerbation was mainly driven by the increase in the number of admissions^12^. Monitoring the 30-day readmission rate using a national or regional representative database is needed to evaluate the care quality and economic burden of COPD patients. The long-term trend pattern of readmission over time is not well characterized in China. Early readmissions may usually be preventable if high-risk patients could be identified^7^. The number and types of comorbidities have been linked to readmission risk among COPD patients^13^, but most studies have been conducted in Europeans and Americans. Common comorbidities of COPD may vary across populations, and it is still unclear whether the association between comorbidities and COPD readmission risk differs between populations.

The present study aimed to describe the secular trend in COPD-related 30-day readmissions in Beijing during 2012–2017 based on a population-based citywide representative data. To identify patients at higher risk of 30-day readmission, we also examine risk factors for COPD readmission and in-hospital mortality (IHM) within 30 days.

## Methods

### Data source

We conducted a retrospective analysis using a hospital discharge database maintained by the Beijing Municipal Health Commission Information Centre. All secondary- and tertiary-level hospitals in Beijing are required to submit a standardized discharge record for each hospitalization. Only secondary- and tertiary-level hospitals admit inpatients in Beijing, therefore the database could capture all hospitalization events in Beijing. The Research Ethics Board of Beijing Chaoyang Hospital approved the study and waived the requirement for informed consent (2018-ke-303). Data were de-identified before analysis. It is impossible to identify patients at an individual level either in this article or in the retrieved database. Given the anonymous and mandatory nature of the data, informed consent was neither required nor necessary. All methods were performed in accordance with the ethical guidelines for human participants.

### Study population

We included patients aged ≥ 40 years who were living in Beijing permanently from 2012 to 2017. During the study period, an index COPD admission was defined as a hospitalization with a primary diagnosis of COPD (International Classification of Diseases 10th Revision, ICD-10: J40-J44) that did not result in in-hospital death. We included bronchitis (J40-J42) and emphysema (J43) because they often co-occurred with COPD, and some COPD patients might be diagnosed as bronchitis or emphysema in practice. We also performed a sensitivity analysis using ICD-10 code J44 to define COPD admissions. If a patient had more than one hospitalization within 30 days, only the first one was counted as an index admission. Discharges against medical advice and those who planned to readmit in 30 days were excluded from index admissions. We excluded December discharges from index admissions because they could not be followed up for 30 days in this year. Finally, a total of 131 591 index admissions were included in our analysis.

### Data collection

We obtained the front page of discharge records from the database. This page included demographic characteristics (age and sex), hospital level (secondary or tertiary), date of admission, length of hospital stay (LOHS) (days), the 10th International Classification of Diseases (ICD-10) codes of primary and other discharge diagnoses, use of mechanical ventilation, medical cost in Chinese yuan (CNY), and discharge outcome (alive or dead). Common comorbidities were determined according to discharge diagnoses. Charlson Comorbidity Index was calculated based on 19 categories of diseases^14^. The frequency of COPD-related hospitalizations (ICD-10: J40-J44) within 1 year prior to the index admission was obtained and categorized into 3 groups: 0, 1, and ≥ 2.

### Outcome

The primary outcome of interest was COPD-related 30-day readmission, defined as the initial admission with a primary diagnosis of COPD (ICD-10: J40-J44) that occurs within 30 days from the discharge date of an index admission. The readmission rate was calculated as the number of index admissions with COPD-related 30-day readmission divided by the total number of index admissions (per 100 index admissions). The secondary outcome was IHM for COPD-related 30-day readmission.

### Statistical analysis

One patient may have multiple hospitalizations, and all statistical analyses were based on hospitalization instead of a person. Characteristics of index admissions and readmissions in each year during the study period were described and compared. Descriptive statistics are presented as means ± standard deviations or medians for continuous variables and as percentages for categorical variables. Annual 30-day readmission rates were calculated in the total population and subgroups defined by age, sex, hospital level, and Charlson index at index admission. Adjusted annual change of 30-day readmission rate was estimated using logistic models with a random effect to account for multiple hospitalizations of one patient^15^. Model covariates included age, sex, hospital level, comorbidity index, and use of mechanical ventilation at index admission. We also described the in-hospital outcome (medical cost, use of mechanical ventilation, LOHS, and IHM) of 30-day COPD readmission by sex and year. We used multivariable logistic models to investigate risk factors for COPD readmission risk and IHM within 30 days. Linear models were used to estimate the association of comorbidities with total costs of the index admission and the 30-day COPD readmission. Statistical analyses were performed using Stata 15.0. All P values were two-sided. Statistical significance was set at < 0.05.

## Results

### Characteristics of index admissions

Between 2012 and 2017, we identified 131 591 index admissions (74 152 patients) submitted by 68 tertiary- and 51 secondary-hospitals, with a primary diagnosis of COPD in Beijing. The mean age of index admissions was 75.2 ± 10.3 years. Compared with 4 011 excluded admissions which occurred in-hospital death, index admissions were more likely to have fewer comorbidities, lower mechanical ventilation rate, and shorter mean LOHS (Table S1). The proportion of men and tertiary-hospital admissions increased over time, and the proportion of mechanical ventilation use showed a decreasing trend (Table S2).

### Trends in COPD-related 30-day readmissions

**Figure 1:**
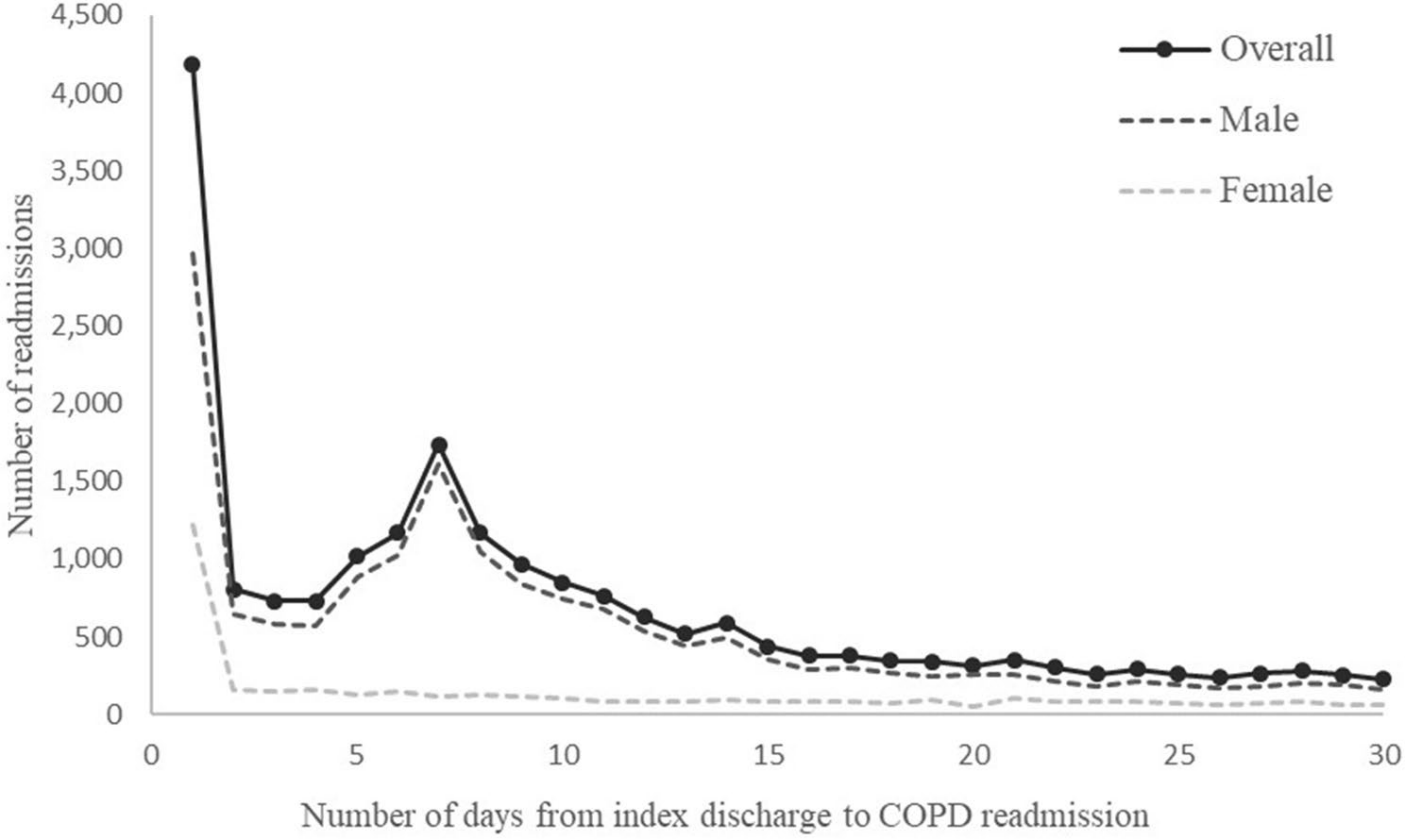
Distribution of time from index discharge to COPD readmission within 30 days.

There were 20 808 COPD-related readmissions within 30 days of index discharge, and the overall 30-day readmission rate was 15.8%. Of all COPD-related readmissions, 20.1% occurred at on first day after index discharge, and 49.9% occurred within 7 days after index discharge. The number of COPD-related readmissions stabilized after day 2 among women, while a readmission peak at day 7 was observed for men (Fig. 1). Table 1 presented characteristics and in-hospital outcomes of readmitted patients by calendar year. Mechanical ventilation use, LOHS, and median medical cost of readmitted patients showed decreasing trends from 2012 to 2017. Female patients were older and were more likely to have comorbidities than male patients. The proportion of mechanical ventilation use and median medical cost were higher in women than in men among patients readmitted to tertiary hospitals (Table S3).

**Table 1:** Characteristics of 30-day readmitted patients during 2012–2017 (n = 20 808).

Variables	Overall	2012	2013	2014	2015	2016	2017	P_trend_
Number of readmissions	20 808	2081	3173	4390	3392	3809	3963	
Readmission rate, %	15.8%	11.5%	13.9%	17.5%	16.9%	17.3%	17.2%	< 0.001
Male, %	80.6	72.1	75.2	81.9	83.3	82.6	83.8	< 0.001
Age (yrs),$\overline{x}\pm \mathrm{SD }$	74.7 ± 10.7	77.0 ± 9.8	75.2 ± 11.0	73.7 ± 11.4	74.4 ± 10.7	74.8 ± 10.3	74.4 ± 10.2	< 0.001
**Age group, %**								< 0.001
40–55 yrs	2.8	3.3	4.8	4.5	1.9	1.3	1.2	
55–65 yrs	18.6	9.3	14.8	22.1	22.1	19.3	18.8	
65–75 yrs	22.2	19.3	18.3	18.8	21.8	25.4	28.3	
75–85 yrs	37.3	47.5	42.9	36.5	34.5	34.3	33.4	
≥ 85 yrs	19.1	20.6	19.2	18.1	19.6	19.7	18.4	
Tertiary Hospital, %	74.6	72.9	78.3	75.5	73.2	73.9	73.3	0.003
**Charlson index, %**								< 0.001
0	30.4	18.5	24.2	32.5	32.9	33.9	34.0	
1	29.2	30.3	30.0	28.6	28.6	28.8	29.4	
2	19.7	24.3	21.1	18.8	19.1	18.4	18.9	
≥ 3	20.7	26.9	24.7	20.1	19.5	18.9	17.7	
Mechanical ventilation, %	5.8	7.2	7.9	7.4	4.7	4.6	3.7	< 0.001
LOHS (days), $\overline{x}$ ± SD	17.8 ± 12.2	19.2 ± 15.2	18.5 ± 13.6	17.9 ± 11.5	18.0 ± 13.2	16.9 ± 11.0	17.2 ± 9.7	< 0.001
Median hospitalization cost (CNY)	13,690	13,792	12,722	11,945	13,580	12,783	12,957	0.001

All costs were adjusted for the increment of inflation.

*LOHS*, length of hospital stay, *CNY* Chinese yuan.

**Figure 2:**
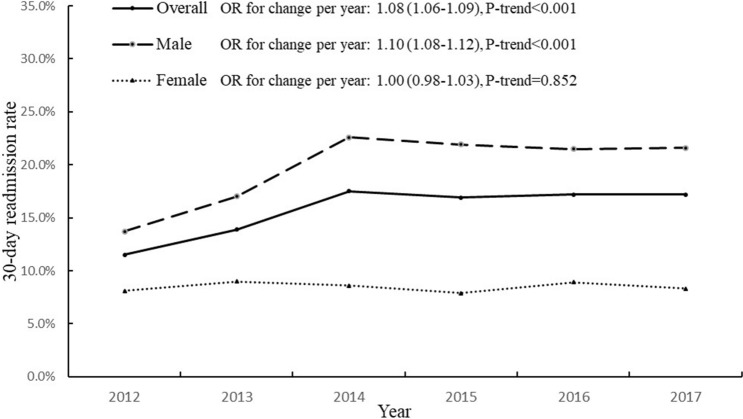
Gender-specific trends in 30-day COPD readmission rates during 2012–2017. ORs (95% CIs) for change in readmission rates per year were calculated using logistic regression models. Covariates included age, sex (for total population), hospital level, Charlson index, length of hospital stay, and use of mechanical ventilation at index admission.

The secular trend in annual COPD-related readmission rate was presented in Fig. 2. Overall, the readmission rate increased from 11.5% in 2012 to 17.2% in 2017 (P_trend_ < 0.001). The adjusted OR (95% CI) for the annual change in the 30-day readmission rate was 1.08 (1.06–1.09) (P_trend_ < 0.001). The readmission rate increased significantly from 2012 to 2014 (11.5% to 17.5%) and then remained almost unchanged at around 17% from 2014 to 2017. Men (19.9%) had a higher readmission rate than women (8.5%), with different trend patterns from women. The change of readmission rate over time in men was similar to the overall trend, with an adjusted OR (95% CI) for annual change to be 1.10 (1.08–1.12) (P_trend_ < 0.001). Whereas in women, the readmission rate did not change significantly during the study period (P_trend_ = 0.852). No significant differences in secular trends were found between subgroups by age, sex, Charlson index, and hospital level (Table S4). When using ICD-10 code J44 to define COPD, a total of 121 149 index admissions were identified, and the overall 30-day readmission rate was 16.3%. The secular trend of 30-day readmission rate under the J44 definition was similar to the main results (Fig. S1).

### In-hospital mortality of 30-day readmissions

Among 20 808 COPD-related readmissions, 709 (3.4%) in-hospital deaths occurred. Women had higher IHM than men (6.0% vs. 2.8%). The crude IHM of 30-day readmission decreased from 4.2% in 2012 to 2.8% in 2017. After multivariable adjustment, the IHM did not change significantly during 2012–2017 (OR for change per year: 0.99, 95% CI 0.93–1.07) (Fig. S2).

### Risk factors for readmission and in-hospital mortality within 30 days

Compared with hospitalizations without 30-day COPD-related readmission, those who readmitted are more likely to be male, hospitalized for COPD in the previous year, from tertiary hospitals at index admission, have a higher proportion of mechanical ventilation, have higher medical costs, and have longer LOHS. The prevalence of common comorbidities differed between those with and without 30-day readmissions (Table 2).

**Table 2:** Comparison of characteristics at index admission between those with and without 30-day COPD readmission (n = 131 591).

Variables	Overall	With readmission	Without readmission	*P-*value
Number	131 591	20 808	110 783	
Male, %	63.9	80.6	60.7	< 0.001
Age (yrs), $\overline{x}$ ± SD	75.2 ± 10.3	74.7 ± 10.7	75.3 ± 10.2	< 0.001
**Age group, %**				< 0.001
40–55 yrs	3.7	2.8	3.9	
55–65 yrs	13.7	18.7	12.7	
65–75 yrs	22.0	22.3	22.0	
75–85 yrs	43.2	37.3	44.2	
≥ 85 yrs	17.4	18.9	17.1	
**Frequency of COPD admissions in previous year**				< 0.001
0	58.0	25.1	64.2	
1	16.7	11.8	17.6	
≥ 2	25.3	63.1	18.2	
Tertiary Hospital, %	69.3	73.5	68.5	< 0.001
**Charlson index, %**				< 0.001
0	26.8	31.1	26.0	
1	32.7	30.3	33.1	
2	21.0	19.0	21.3	
≥ 3	19.6	19.6	19.6	
**Comorbidities, %**				
Hypertension	57.5	58.6	57.2	< 0.001
Diabetes	21.2	20.7	21.3	0.073
Osteoporosis	4.2	4.9	4.1	< 0.001
Coronary heart disease	49.6	53.8	48.8	< 0.001
Cerebral vascular disease	27.1	26.6	27.2	0.099
Congestive heart failure	16.4	15.4	16.6	< 0.001
Anxiety or depression	2.5	2.6	2.5	0.159
Cancer	4.3	4.5	4.3	0.099
Mechanical ventilation, %	3.5	5.3	3.1	< 0.001
LOHS (days), $\overline{x}$ ± SD	13.6 ± 10.1	18.2 ± 16.2	12.8 ± 8.2	< 0.001
Median hospitalization cost (CNY)	12,843	13,202	12,750	< 0.001

The unit of analysis was hospitalization instead of patient. All costs were adjusted for the increment of inflation.

*LOHS* length of hospital stay, *CNY* Chinese yuan.

Multivariable analyses showed that risk factors collected at index admission for 30-day readmission risk included male (OR = 1.89, 95% CI 1.78–2.00), hospitalized for COPD in the previous year, admitted to tertiary-hospital (OR = 1.10, 95% CI 1.04–1.16), Charlson index ≥ 3 (OR = 1.09, 95% CI 1.01–1.16), mechanical ventilation use (OR = 1.74, 95% CI 1.57–1.92), LOHS longer than 10 days, and having the following comorbidities: osteoporosis (OR = 1.14, 95% CI 1.02–1.27), coronary heart disease (OR = 1.16, 95% CI 1.10–1.22), congestive heart failure (OR = 1.09, 95% CI 1.02–1.16), and cancer (OR = 1.29, 95% CI 1.16–1.44) (Table 3). Using ICD-10 code J44 to define COPD did not change the results substantially (Table S5). Similar results were observed for IHM within 30 days (Table S6). Comorbid diabetes, coronary heart disease, cerebral vascular disease, congestive heart failure, and cancer were associated with 900 (642–1 158) CNY, 1 288 (1 076–1 500) CNY, 1 589 (1 359–1 818) CNY, 3 427 (3 161–3 693) CNY, and 2 392 (1 902–2 882) CNY higher costs of the index admission and 30-day COPD readmission, respectively (Table S7).

**Table 3:** Multivariable analysis of factors at index admission associated with 30-day COPD readmission risk (n = 131 591).

Variables	Age and sex adjusted		Multivariable adjusted	
	OR (95% CI)	*P*-value	OR (95% CI)	*P*-value
Male (vs. female)	2.42 (2.27–2.57)	< 0.001	1.89 (1.78–2.00)	< 0.001
Age ≥ 65 yrs (vs. < 65 yrs)	1.33 (1.24–1.44)	< 0.001	1.04 (0.97–1.12)	0.227
Admission year (per year)	1.08 (1.06–1.09)	< 0.001	1.03 (1.01–1.04)	< 0.001
**Frequency of COPD admissions in previous year (vs. 0)**				
1	2.27 (2.14–2.41)	< 0.001	2.08 (1.96–2.21)	< 0.001
≥ 2	5.67 (5.38–5.97)	< 0.001	4.96 (4.72–5.23)	< 0.001
Hospital level (Tertiary vs. Secondary)	1.23 (1.16–1.31)	< 0.001	1.10 (1.04–1.16)	0.001
**Charlson index (vs. 0)**				
1	1.09 (1.02–1.16)	0.013	0.96 (0.90–1.02)	0.156
2	1.28 (1.19–1.37)	< 0.001	0.98 (0.91–1.05)	0.516
≥ 3	1.55 (1.43–1.67)	< 0.001	1.09 (1.01–1.16)	0.019
Use of mechanical ventilation (vs. not use)	2.45 (2.20–2.73)	< 0.001	1.74 (1.57–1.92)	< 0.001
**Length of hospital stay (vs. ≤ 10 days)**				
11–14 days	1.34 (1.26–1.42)	< 0.001	1.28 (1.21–1.35)	< 0.001
> 14 days	3.30 (3.12–3.49)	< 0.001	2.95 (2.80–3.11)	< 0.001
**Comorbidities (presence vs. absence)**				
Hypertension	1.06 (1.00–1.12)	0.037	1.01 (0.96–1.06)	0.780
Diabetes	1.04 (0.97–1.11)	0.244	0.95 (0.89–1.01)	0.091
Osteoporosis	1.44 (1.28–1.62)	< 0.001	1.14 (1.02–1.27)	0.019
Coronary heart disease	1.44 (1.37–1.52)	< 0.001	1.16 (1.10–1.22)	< 0.001
Cerebral vascular disease	1.12 (1.06–1.19)	< 0.001	1.05 (0.99–1.11)	0.070
Congestive heart failure	1.38 (1.29–1.47)	< 0.001	1.09 (1.02–1.16)	0.007
Anxiety or depression	1.36 (1.17–1.58)	< 0.001	1.13 (0.99–1.30)	0.078
Cancer	1.56 (1.39–1.75)	< 0.001	1.29 (1.16–1.44)	< 0.001

ORs were estimated using logistic models with a random effect to account for multiple hospitalizations of one patient. Multivariable model included age, sex, hospital level, length of hospital stay, Charlson index, use of mechanical ventilation, the frequency of COPD admissions in the previous year, and admission year at index admission.

## Discussion

In this 6-year trend analysis of a city-wide representative database in Beijing, we found that the overall 30-day readmission rate for COPD increased at an adjusted annual rate of 8% during 2012–2017. The readmission rate of men was higher and increased faster than women. Having comorbid osteoporosis, coronary heart disease, congestive heart failure, and cancer were associated with increased risk of 30-day COPD-related readmission.

As a result of poor inpatient care and discharge management, early readmissions frequently occur in COPD patients. An analysis of the US Health Cost and Utilization Project (HCUP) database found that 18.9% of COPD index hospitalizations had at least one 30-day readmission in 2013^16^. Another study in London reported a 30-day readmission rate of 10.2% between 2006 and 2010^9^. In the present study, we restricted the analysis to COPD-related readmissions (defined by primary diagnosis) and found that the overall 30-day readmission rate was 15.8% in Beijing between 2012 to 2017. Variability in rates of COPD readmissions between countries might be explained by differences in study methodology, ethnicity, disease diagnosis, and care quality. Nearly a half of 30-day COPD readmissions occurred within 1 week after discharge in our study. Previous studies also found that most readmissions occurred on the first day after discharge^16,17^. Early readmissions were thought to be more preventable and amenable to hospital-based interventions^18^. The relatively high readmission rates and a large proportion of early readmissions indicated the need for improving the quality of care and treatment received during and immediately after hospitalization.

Reducing readmissions for acute exacerbation of COPD has become a global challenge. COPD readmission within 30 days is associated with a higher economic burden and subsequent mortality risk^19,20^. In response, several countries developed policies linking payments to reducing readmissions. For example, the US Hospital Readmissions Reduction Program (HRRP) penalized hospitals with excess readmissions for targeted conditions including COPD^21^. Policy-related interventions to reduce hospital readmissions have shown variable temporal trends in 30-day readmission rates after COPD hospitalization in different countries. The 30-day readmission rate showed a modest decline trend from 2006 to 2014 in the US^22–24^. During the same period, a Spanish study based on a national discharge database found a significant reduction in COPD readmissions from 2006 to 2012^25^. By contrast, the 30-day readmission rate in Beijing increased significantly from 11.5% in 2012 to 17.2% in 2017. The upward trend in readmission rate and the accompanying downward trend in IHM might partly reflect better access to hospital care and better treatment modalities during hospitalization for COPD in China. In addition, the severity of inpatients with COPD declined, which might also result in better survival rates^26^. But readmissions are responsible for most of the expenses related to the disease^27,28^, and many of them could be prevented with strategies to improve the quality of care^29^.

Moreover, early readmission rates for COPD could be reduced by some interventions including early follow-up care and care bundle after hospital discharge^30^. The care bundle includes continuity of care with a primary care physician or pulmonologist, optimization of medication, supervision of inhaler use, assessment and management of comorbidities, smoking cessation, and referral to pulmonary rehabilitation^4^. In addition, COPD self-management is essential in preventing readmissions, especially for high-risk subgroups such as female and older patients. Future research could evaluate the application of the COPD care bundle in China.

We observed that the 30-day readmission rate levelled off from 2014 to 2017 in Beijing. Several factors may contribute to this stable trend. First, the regional integrated care alliances were established in Beijing in 2013. To improve the quality and efficiency of healthcare services, the Beijing Municipal Health Commission promoted collaboration between secondary/tertiary hospitals and primary care institutions. Since 2013, a mutual referral system was gradually established across the city. Under this system, patients would seek primary care first before hospitalization, and inpatients would be referred to the primary health care institutions to receive subsequent treatment once their conditions are stable^31^. Second, the stable trend in readmission rate since 2014 might be benefited from improvements in air quality in Beijing during 2013–2017 as a result of the Air Pollution Prevention and Control Action Plan (APPCAP)^32^. Third, the decline in smoking prevalence after the implementation of the Beijing Tobacco Control Regulation in 2015 may also partly have contributed to the stable trend^33^.

Identifying patient-level risk factors is important in preventing COPD-related readmissions. LOHS, Charlson index, and use of mechanical ventilation are proxies of disease severity, and they have been used to predict 30-day readmission risk^13^. But limited and inconsistent results have been reported in the Chinese population^34,35^. We confirmed these predictors using a larger database that virtually covers all admissions in Beijing. These factors were also associated with increased risk of IHM at 30-day readmission. In concordance with previous results^36^, we observed a higher risk of 30-day readmission among male patients. But female readmitted patients appeared to be more severe and presented higher IHM than male readmitted patients. This finding may reflect gender disparities in disease phenotype, treatment, and management of COPD^37^. We should pay attention to improving healthcare services and reducing IHM in female readmitted patients.

COPD often coexists with other diseases, particularly in the elderly. Comorbidity is associated with poor outcomes of COPD. Congestive heart failure, osteoporosis, and cancer are established risk factors for exacerbation readmission^13^, and the results hold in this Chinese population. The association between comorbid coronary heart disease (CHD) and COPD-related readmission risk was still not clear. A large study in Canada found that ischaemic heart disease only predicted 1-year COPD readmission in women^38^. Other smaller studies reported inconsistent findings^35,39^. In our study, the prevalence of CHD in COPD hospitalized patients was high (49.6%), and patients with CHD were at higher risk of 30-day readmission. Interventions to reduce COPD readmissions need to target high-risk subgroups identified by comorbidities. In consistent with previous studies^40,41^, we also found that many comorbidities were associated with significantly higher costs in COPD inpatients. The prevention and management of comorbidities may contribute to reducing the economic burden of COPD.

The major strength of this study is the large representative sample in Beijing, the capital of China. Hospitalization information was collected from an administrative database, and the data quality had been strictly controlled by the government. The readmission plan was available on the front page of the discharge record since 2012, which facilitated us to exclude planned readmissions within 30 days. There are also several limitations. First, the findings of readmission rates and trends could not be generalized to other parts of China because of regional variations in disease spectrum and healthcare quality. Second, some covariates were not available, such as smoking status, medication use, lung function, and severity of COPD exacerbation. These factors were also associated with readmission risk^36^. Third, COPD-related readmissions could not capture symptom-based exacerbations, which may also reflect the quality of care. However, hospital readmission could be more accurately assessed using a discharge database.

In conclusion, the 30-day COPD-related readmission rate in Beijing showed an overall increasing trend from 2012 to 2017. Comorbid conditions were associated with the risk of 30-day readmission. These findings can guide future efforts to improve hospital treatment and post-discharge management in COPD patients. Preventing early readmissions after COPD hospitalizations would help reduce the significant disease burden in China.

## Supplementary Information

Supplementary Information.

## Data Availability

The data that support the findings of this study are available from Beijing Municipal Health Commission Information Centre but restrictions apply to the availability of these data, which were used under license for the current study, and so are not publicly available. Data are however available from the authors upon reasonable request and with permission of Beijing Municipal Health Commission Information Centre.
