# Supplementary material for: Secular trend and risk factors of 30-day COPD-related readmission in Beijing, China
Source: Sci Rep. 2022 Oct 5;12:16589. doi: 10.1038/s41598-022-20884-3 (PMC9534919; doi:10.1038/s41598-022-20884-3)
Supplement: Supplementary file 1 — Supplementary Information. [file 41598_2022_20884_MOESM1_ESM.pdf]

Secular trend and risk factors of 30-day COPD-related readmission in Beijing, China

Supplementary Appendix

Table of Contents

Table S1. Comparison of baseline characteristics between index admissions and excluded admissions due to in-hospital mortality

Table S2. Characteristics of index admissions by calendar years

Table S3. Differences in characteristics at 30-day COPD readmission between men and women during 2012-2017

Table S4. Trends in 30-day COPD readmission rates by Subgroups during 2012-2017

Figure S1. Secular trend in 30-day COPD readmission rates during 2012-2017 under the J44 definition of COPD

Figure S2. Trend in in-hospital mortality rate of COPD readmission during 2012-2017

Table S5. Multivariable analysis of factors at index admission associated with 30-day COPD readmission risk under the ICD-10 J44 definition

Table S6. Predictors at index admission of COPD related 30-day in-hospital mortality

Table S7. The association between comorbidities and total costs of the index admission and 30-day COPD readmission

Table S1. Comparison of baseline characteristics between index admissions and excluded admissions due to in-hospital mortality

Variables	Index admissions	Excluded admissions	*P*-value
Number	131 591	4 011	
Gender, %			<0.001
Male	63.9	59.2	
Female	36.1	40.8	
Age (yrs), $\bar{x} \pm SD$	75.2±10.3	82.4±7.5	<0.001
Hospital level, %			0.123
Secondary	30.7	29.6	
Tertiary	69.3	70.4	
Charlson index, %			<0.001
0	26.8	6.9	
1	32.7	20.8	
2	21.0	25.4	
⩾3	19.5	46.9	
Mechanical ventilation, %	3.5	30.3	<0.001
LOHS (days), $\bar{x} \pm SD$	13.6±10.1	17.7±28.3	<0.001

Note: LOS, length of hospital stay.

Table S2. Characteristics of index admissions by calendar years

Variables	2012	2013	2014	2015	2016	2017	P_{trend}
Number	19662	22605	25481	19941	22135	21767	
Gender, %							<0.001
Male	60.6	61.0	64.0	64.4	66.4	66.7	
Female	39.4	39.0	36.0	35.6	33.6	33.3	
Age (yrs), $\bar{x} \pm SD$	75.3±10.0	75.3±10.3	74.9±10.5	75.1±10.4	75.2±10.3	75.4±10.3	0.457
Age group, %							<0.001
40-55 yrs	4.1	4.2	4.2	3.5	3.3	3.0	
55-65 yrs	11.1	12.7	14.6	14.8	14.6	14.0	
65-75 yrs	22.3	20.3	21.0	22.0	23.2	23.8	
75-85 yrs	47.3	46.1	43.2	42.0	40.6	40.0	
⩾85 yrs	15.3	16.6	17.0	17.7	18.4	19.2	
Hospital level, %							<0.001
Secondary	32.3	31.2	31.0	30.3	30.3	29.3	
Tertiary	67.7	68.8	69.0	69.7	69.7	70.7	
Charlson index, %							<0.001
0	26.7	25.9	27.8	27.7	26.7	25.9	
1	33.4	33.5	33.3	31.3	32.0	32.3	
2	21.0	21.0	20.0	21.4	21.2	21.4	
⩾3	18.9	19.6	18.9	19.6	20.0	20.4	
Mechanical ventilation, %	3.7	4.0	4.1	3.1	3.0	2.8	<0.001
LOHS (days), $\bar{x} \pm SD$	14.4±13.1	13.8±12.7	13.9±8.6	13.5±9.2	13.2±8.1	13.0±7.7	<0.001
Median hospitalization cost (CNY)	12002	12365	12553	13246	13458	13415	<0.001

Note: LOHS, length of hospital stay. CNY, Chinese yuan.

Table S3. Differences in characteristics at 30-day COPD readmission between men and women during 2012-2017

Characteristics	Secondary hospital			Tertiary hospital		
	Men	Women	*P*	Men	Women	*P*
Number	4 057	1 237		1 2716	2 798	
Age (yrs), $\bar{x} \pm$ SD	75.0±10.2	79.0±8.3	<0.001	73.0±11.0	80.3±7.9	<0.001
Charlson index, %			<0.001			<0.001
0	35.2	12.9		34.2	14.2	
1	26.9	29.2		30.2	27.8	
2	21.5	29.3		17.0	24.8	
≥ 3	16.4	28.6		18.5	33.3	
Mechanical ventilation, %	5.1	5.6	0.488	5.1	9.9	<0.001
LOHS (days), M[IQR]	16.8±13.4	18.7±20.3	<0.001	18.4±10.0	16.5±14.0	<0.001
Median hospitalization cost (CNY)	11910	12220	0.558	12810	16889	<0.001
Death, %	2.7	6.0	<0.001	2.8	6.0	<0.001

Note: LOHS, length of hospital stay; CNY, Chinese yuan.

Table S4. Trends in 30-day COPD readmission rates by Subgroups during 2012-2017

	2012	2013	2014	2015	2016	2017	Adjusted OR (95%CI) for annual change in readmission rate	P_{trend}	*P* for interaction
Overall	11.5%	13.9%	17.5%	16.9%	17.3%	17.2%	1.08 (1.06-1.09)	<0.001	
Sex									<0.001
Male	13.7%	17.0%	22.6%	21.9%	21.5%	21.7%	1.10 (1.08-1.12)	<0.001	
Female	8.1%	9.0%	8.6%	7.9%	8.9%	8.3%	1.00 (0.98-1.03)	0.852	
Age									0.305
< 65 yrs	9.8%	15.9%	25.3%	22.3%	20.1%	20.3%	1.07 (1.03-1.11)	0.001	
≥ 65 yrs	11.8%	13.5%	15.7%	15.7%	16.6%	16.6%	1.08 (1.06-1.09)	<0.001	
Hospital level									0.477
Secondary	10.5%	10.3%	14.3%	15.6%	15.3%	16.0%	1.06 (1.04-1.09)	<0.001	
Tertiary	12.0%	15.5%	19.0%	17.5%	18.1%	17.7%	1.08 (1.07-1.10)	<0.001	
Charlson index									0.079
0	8.9%	13.3%	21.0%	20.9%	21.9%	22.7%	1.12 (1.08-1.16)	<0.001	
1	11.2%	13.0%	15.8%	15.6%	15.6%	16.5%	1.08 (1.06-1.11)	<0.001	
2	12.2%	13.7%	15.7%	15.2%	14.8%	14.2%	1.06 (1.03-1.09)	0.009	
≥ 3	14.9%	16.4%	17.4%	15.3%	16.2%	14.4%	1.02 (0.99-1.05)	0.184	

Note: Annual change of readmission rates were estimated using logistic models with a random effect to account for multiple hospitalizations of one patient. Model adjusted for age, sex, hospital level, Charlson index, length of stay, and use of mechanical ventilation at index admission.

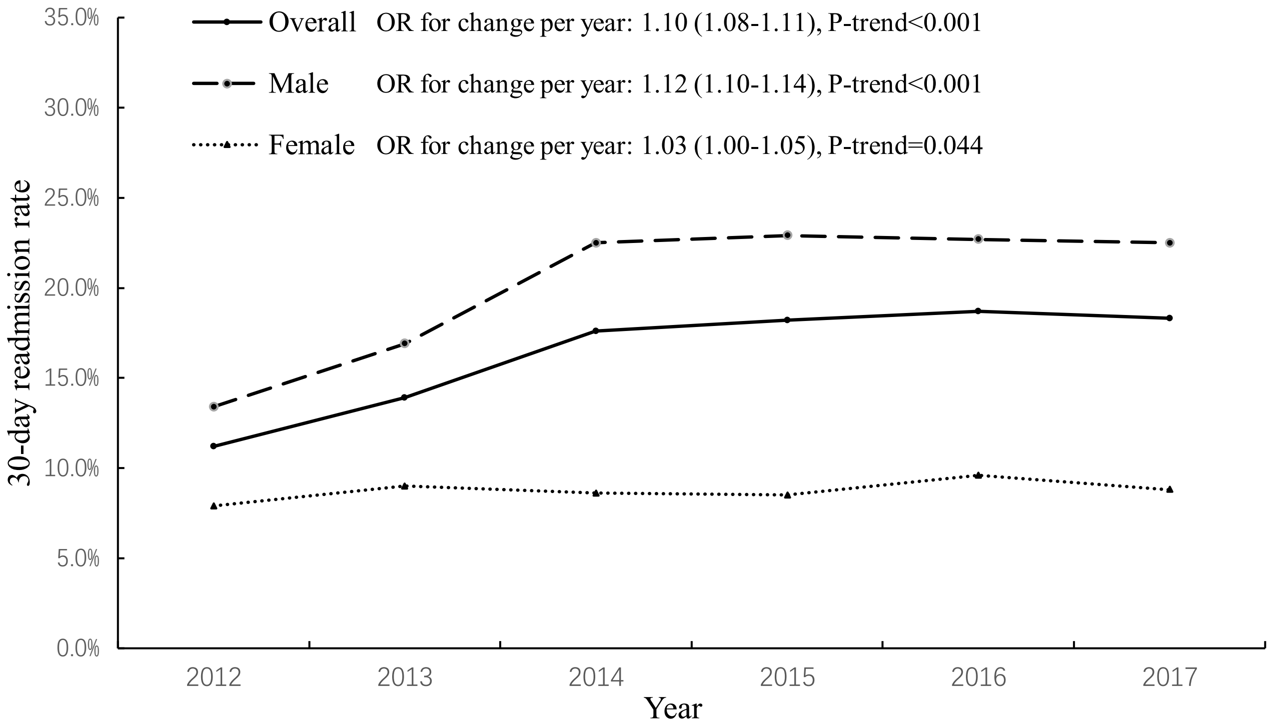

Figure S1. Secular trend in 30-day COPD readmission rates during 2012-2017 under the J44 definition of COPD. ORs (95% CIs) for change in readmission rates per year were calculated using logistic regression models. Covariates included age, sex (for total population), hospital level, Charlson index, length of hospital stay, and use of mechanical ventilation at index admission.

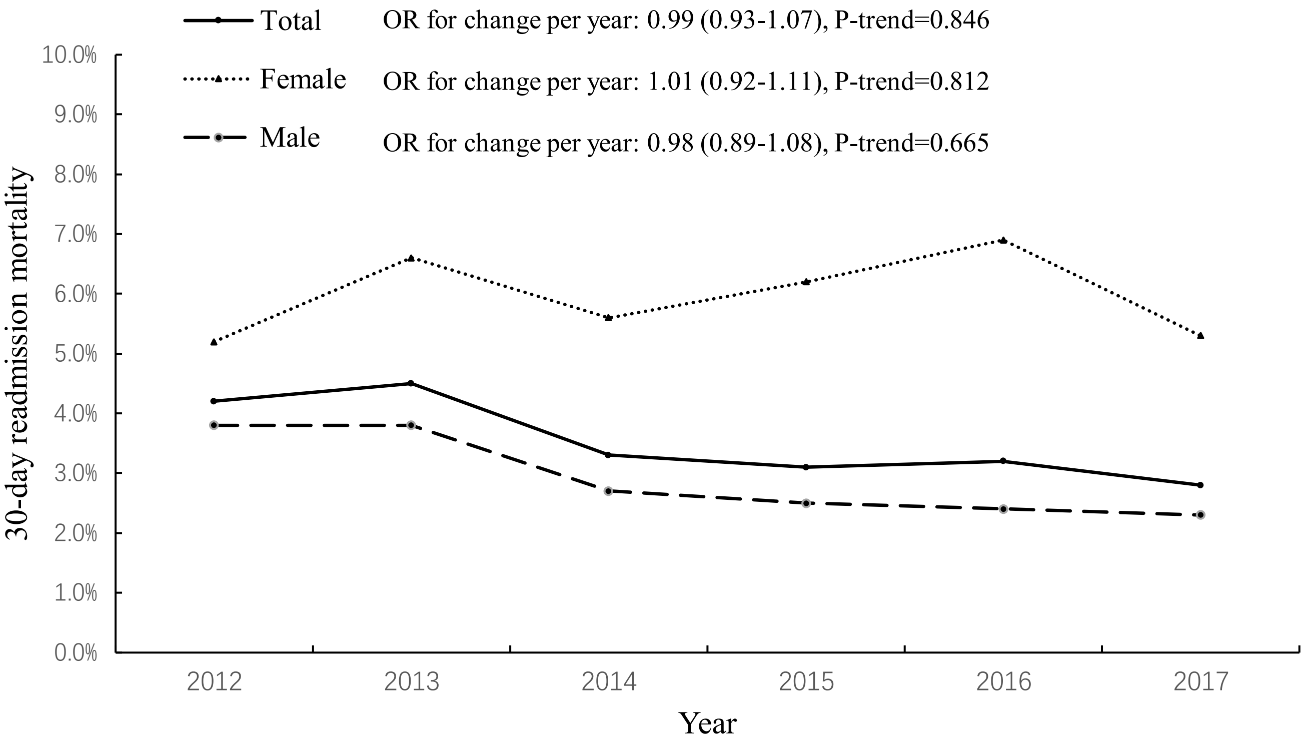

Figure S2. Trend in in-hospital mortality rate of COPD readmission during 2012-2017 (n=20 808). ORs (95% CIs) for change in mortality rates per year were calculated using logistic regression models. Covariates included age, sex (for total population), hospital level, Charlson index, length of hospital stay, and use of mechanical ventilation at the readmission.

Table S5. Multivariable analysis of factors at index admission associated with 30-day COPD readmission risk under the ICD-10 J44 definition (n=121 149)

Variables	Age and sex adjusted		Multivariable adjusted	
	OR (95% CI)	*P*-value	OR (95% CI)	*P*-value
Male (vs. female)	2.27 (2.13-2.43)	<0.001	1.83 (1.73-1.94)	<0.001
Age ≥ 65 yrs (vs. <65 yrs)	1.04 (0.96-1.12)	0.354	0.89 (0.83-0.96)	0.002
Admission year (per year)	1.09 (1.08-1.11)	<0.001	1.04 (1.03-1.05)	<0.001
Frequency of COPD admissions in previous year (vs. 0)				
1	2.12 (1.99-2.26)	<0.001	1.98 (1.86-2.11)	<0.001
⩾2	5.12 (4.85-5.39)	<0.001	4.58 (4.35-4.83)	<0.001
Hospital level (Tertiary vs. Secondary)	1.17 (1.09-1.24)	<0.001	1.07 (1.01-1.13)	0.024
Charlson index (vs. 0)				
1	1.03 (0.96-1.10)	0.454	0.92 (0.86-0.97)	0.006
2	1.14 (1.05-1.23)	0.001	0.90 (0.84-0.96)	0.002
≥ 3	1.28 (1.19-1.39)	<0.001	0.94 (0.87-1.00)	0.067
Use of mechanical ventilation (vs. not use)	1.65 (1.49-1.83)	<0.001	1.25 (1.14-1.38)	<0.001
Length of hospital stay (vs. ⩽10 days)				
11-14 days	1.34 (1.26-1.43)	<0.001	1.26 (1.19-1.34)	<0.001
> 14 days	3.12 (2.94-3.30)	<0.001	2.67 (2.53-2.81)	<0.001
Comorbidities (presence vs. absence)				
Hypertension	1.09 (1.03-1.15)	0.002	1.05 (1.00-1.10)	0.059
Diabetes	1.06 (0.99-1.13)	0.108	1.01 (0.95-1.08)	0.764
Osteoporosis	1.48 (1.32-1.67)	<0.001	1.18 (1.06-1.32)	0.003
Coronary heart disease	1.39 (1.32-1.47)	<0.001	1.16 (1.10-1.22)	<0.001
Cerebral vascular disease	1.10 (1.04-1.17)	0.002	1.07 (1.01-1.14)	0.018

Variables	Age and sex adjusted		Multivariable adjusted	
	OR (95% CI)	*P*-value	OR (95% CI)	*P*-value
Congestive heart failure	1.19 (1.12-1.27)	<0.001	1.03 (0.96-1.10)	0.396
Anxiety or depression	1.37 (1.18-1.60)	<0.001	1.14 (0.99-1.32)	0.065
Cancer	1.41 (1.26-1.58)	<0.001	1.26 (1.12-1.40)	<0.001

Note: ORs were estimated using logistic models with a random effect to account for multiple hospitalizations of one patient. Multivariable model included age, sex, hospital level, length of hospital stay, Charlson index, use of mechanical ventilation, the frequency of COPD admissions in the previous year, and admission year at index admission.

Table S6. Predictors at index admission of COPD related 30-day in-hospital mortality (n=131 591)

Variables	Age and sex adjusted		Multivariable adjusted	
	OR (95% CI)	*P*-value	OR (95% CI)	*P*-value
Male (vs. female)	1.20 (1.03-1.40)	0.022	1.09 (0.93-1.29)	0.290
Age ≥ 65 yrs (vs. <65 yrs)	8.36 (5.23-13.36)	<0.001	6.35 (3.92-10.29)	<0.001
Admission year (per year)	0.96 (0.92-1.01)	0.119	0.98 (0.93-1.03)	0.399
Frequency of COPD admissions in previous year (vs. 0)				
1	1.31 (1.05-1.64)	0.018	1.13 (0.91-1.03)	0.271
⩾2	2.64 (2.09-3.34)	<0.001	1.86 (1.50-2.29)	<0.001
Hospital level (Tertiary vs. Secondary)	1.18 (1.00-1.39)	0.054	1.02 (0.85-1.21)	0.859
Charlson index (vs. 0)				
1	1.94 (1.45-2.59)	<0.001	1.87 (1.39-2.52)	<0.001
2	3.28 (2.47-4.37)	<0.001	2.96 (2.20-3.98)	<0.001
≥ 3	5.13 (3.90-6.75)	<0.001	4.36 (3.27-5.81)	<0.001
Use of mechanical ventilation (vs. not use)	3.86 (3.08-4.84)	<0.001	2.62 (2.04-3.37)	<0.001
Length of hospital stay (vs. ⩽10 days)				
11-14 days	1.13 (0.88-1.46)	0.330	1.13 (0.89-1.44)	0.298
> 14 days	4.06 (3.34-4.93)	<0.001	3.48 (2.83-4.28)	<0.001
Comorbidities (presence vs. absence)				
Hypertension	0.78 (0.67-0.91)	0.001	0.68 (0.58-0.79)	<0.001
Diabetes	0.91 (0.76-1.10)	0.321	0.57 (0.47-0.70)	<0.001
Osteoporosis	0.84 (0.57-1.25)	0.391	0.68 (0.45-1.03)	0.068
Coronary heart disease	1.57 (1.35-1.84)	<0.001	1.27 (1.08-1.50)	0.004
Cerebral vascular disease	1.28 (1.10-1.50)	0.002	0.86 (0.73-1.03)	0.096
Congestive heart failure	2.55 (2.18-2.98)	<0.001	1.58 (1.32-1.89)	<0.001

Variables	Age and sex adjusted		Multivariable adjusted	
	OR (95% CI)	*P*-value	OR (95% CI)	*P*-value
Anxiety or depression	1.38 (0.92-2.06)	0.116	1.31 (0.85-2.00)	0.218
Cancer	3.09 (2.48-3.85)	<0.001	2.06 (1.60-2.65)	<0.001

Note: ORs were estimated using logistic models with a random effect to account for multiple hospitalizations of one patient. Multivariable model included age, sex, hospital level, length of hospital stay, comorbidity index, use of mechanical ventilation, the frequency of COPD admissions in the previous year, and admission year at index hospitalization.

Table S7. The association between comorbidities and total costs of the index admission and 30-day COPD readmission

Comorbidities (with vs. without)	β (95% CI) for total costs, Chinese Yuan	*P* value
Hypertension	-57 (-269-156)	0.600
Diabetes	900 (642-1158)	<0.001
Osteoporosis	341 (-161-844)	0.183
Coronary heart disease	1288 (1076-1500)	<0.001
Cerebral vascular disease	1589 (1359-1818)	<0.001
Congestive heart failure	3427 (3161-3693)	<0.001
Anxiety or depression	405 (-235-1046)	0.215
Cancer	2392 (1902-2882)	<0.001

Note: Total costs were calculated as the costs of the index admission plus the costs of the 30-day COPD readmission (0 if no 30-day COPD readmission). β (95% CI) for each comorbidity was estimated using linear model with a random effect to account for multiple hospitalizations of one patient. Covariates included age, sex, hospital level, length of hospital stay, use of mechanical ventilation, the frequency of COPD admissions in the previous year, and admission year at index hospitalization.
